# Effects of four bolete species on ectomycorrhizae formation and development in *Pinus thunbergii* and *Quercus acutissima*

**DOI:** 10.1186/s12862-024-02239-w

**Published:** 2024-04-25

**Authors:** Qianwen Tan, Lunhe You, Chen Hao, Jianrui Wang, Yu Liu

**Affiliations:** 1 School of Agriculture, Ludong University, Yan Tai, China; 2 Plant Stem Cell Research Institute, Shandong Anran Nanometer Industrial Development Co., Ltd, Hangzhou, Weihai China

**Keywords:** Plant hormones, Arboriculture, Symbiosis, Forest health, *Suillus bovinus*, *Suillus luteus*, *Suillus grevillei*, *Reitboletus sinensis*

## Abstract

**Background:**

Bolete cultivation is economically and ecologically valuable. Ectomycorrhizae are advantageous for plant development and productivity. This study investigated how boletes affect the formation of *Pinus thunbergii* and *Quercus acutissima* ectomycorrhizae using greenhouse-based mycorrhizal experiments, inoculating *P. thunbergii* and *Q. acutissima* with four species of boletes (*Suillus bovinus*, *Suillus luteu*s, *Suillus grevillei*, and *Retiboletus sinensis*).

**Results:**

Three months after inoculation, morphological and molecular analyses identified *S. bovinus*, *S. luteu*s, *S. grevillei* and *R. sinensis* ectomycorrhizae formation on the roots of both tree species. The mycorrhizal infection rate ranged from 40 to 55%. The host plant species determined the mycorrhiza morphology, which was independent of the bolete species. Differences in plant growth, photosynthesis, and endogenous hormone secretion primarily correlated with the host plant species. Infection with all four bolete species significantly promoted the host plants’ growth and photosynthesis rates; indole-3-acetic acid, zeatin, and gibberellic acid secretion increased, and the abscisic acid level significantly decreased. Indole-3-acetic acid was also detected in the fermentation broths of all bolete species.

**Conclusions:**

Inoculation with bolete and subsequent mycorrhizae formation significantly altered the morphology and hormone content in the host seedlings, indicating growth promotion. These findings have practical implications for culturing pine and oak tree species.

## Background

Ectomycorrhizal fungi (ECMFs) are widespread in forest systems and have existed for 125 million years. However, ECMFs form ectomycorrhizae in only 3% of vascular plants. Nevertheless, they have effectively boosted the growth of various tree species, such as pine and oak, contributing to overall forest health [1]. ECMF diversity also affects ecosystem function and plant diversity [2, 3]. Under natural conditions, plants increase their photosynthetic rate in response to the high carbohydrate demand of mycorrhizal symbionts, whereas symbiotic plants also control or limit the flux of photosynthetic products transported to the mycorrhiza, preventing the parasitic effects of ectomycorrhizal bacteria [4].

Ectomycorrhizae are formed when the mycelium infects the roots of the host plant at a relatively early growth stage before the saplings become woody. A mantle is formed on the surface of the root tip, and a Hartig network forms between the root cortex cells [5]. Ectomycorrhizae play an important role in plant growth and development, vegetation restoration, and plant productivity, improving their abilities to absorb nutrients and withstand drought, salt and alkaline conditions, diseases, pests, and heavy metal exposure [6].

Edible mycorrhizal fungi are a subset of ECMF characterised by their edible sporocarps. These fungi are crucial to ecological restoration and support local economies [7]. In some regions of China with abundant forest resources, selling these wild edible fungi can account for more than half of the local residents income. In some cases, mushroom-based ecosystems provide far greater economic benefits than traditional wood industries [8], for example, the sale of *Boletus edulis* [9]. Edible mycorrhizal fungi taste good and are an important source of minerals and non-meat amino acids.

Plant hormones, trace organic compounds synthesised by the plants themselves, produce physiological effects at their synthesis sites or are transported to other parts of the plant, which is crucial for regulating plant growth and development. The infection of host plant roots by ectomycorrhizae and the establishment of direct or indirect symbiotic relationships induce the synthesis of multiple hormones [10], including gibberellins, auxins, cytokinins, ethylene, abscisic acid, brassinosteroids, jasmonic acid, and strigolactones. Owing to the synergistic and antagonistic effects of various hormones, the mechanisms of establishing mycorrhizal symbiosis are complex; each hormone is produced in a dynamic equilibrium state, resulting in growth regulation and normal development of the plant. Previous studies have shown that ECMFs secrete indole compounds [11]. Moreover, ECMFs, such as *Laccaria bicolor*, *Tuber borchii*, and *Tuber melanosporum*, produce a significant amount of the auxin indole-3-acetic acid (IAA), causing morphological changes in symbiotic plant roots. This change results from direct contact between ECMF and the plant’s roots or indirect diffusion signals from the fungi [12, 13]. In mycorrhizal symbionts of poplar trees, fungal hyphae inhibit the growth of the main roots of symbiotic plants and increase the growth of the lateral roots. This effect is similar to that of the exogenous auxin treatment of plant roots [12, 13]. In addition to auxins, *L. bicolor* produces ethylene, which activates the plant auxin synthesis pathway. Ethylene production by ECMFs may induce auxin production in symbiotic plants, enhancing the effects of auxins on root development, particularly by promoting lateral root formation, and new lateral root formation can lead to the development of additional mycorrhizal fungi.

Mycorrhizal synthesis is the first step in developing a cultivation program and determining the optimal host plant-edible mycorrhizal fungi combinations [14]. The traditional and most popular method of raising seedlings in China involves adding fruiting body isolates (mixed, dumped, or injected) to the substrate before or after sowing [15, 16].

An investigation of ectomycorrhizal resources in the Shandong Peninsula, China, resulted in three widely distributed types of boletes, *Suillus bovinus*, *Suillus luteus*, and *Suillus grevillea*, which were also the primary wild edible fungi consumed by the local people. Furthermore, *Retiboletus sinensis* is a newly discovered edible species found in the Shandong Province. These four species of boletes have important economic and ecological value and form symbiotic relationships with pine [17–21]. Furthermore, most of these mushrooms were in the mixed forest, where the predominant oak and pine trees were *Pinus thunbergii* and *Quercus acutissima*. This study explored the effects of these four edible boletes on the growth of symbiotic plants and mycorrhiza formation in *P. thunbergii* and *Q. acutissima*, which could improve the cultivation of pine and oak in this region of China.

## Methods

### Mycelia culture

The four fungal strains were isolated from fruiting bodies using a tissue isolation method (Table 1) and maintained by subculturing every 2–3 months using modified nutrient catabolite agar (MNC) or modified potato glucose agar (PDA) [22, 23]. The modified PDA medium contained 200 g/L of potato, 20 g/L of glucose, 7.88 g/L of peptone, 1 g/L of K_2_HPO_4_, 0.9 g/L of MgSO_4_, 0.05 g/L of CaCl_2_, 0.075 g/L of NaCl, 16.77 g/L of *β*-cyclodextrin, and 0.0055 g/L of ascorbic acid. The modified MNC medium contained 1 g/L of KH_2_PO_4_, 0.5 g/L of MgSO_4_·7H_2_O, 0.5 mL of 0.2% ZnSO_4_, 0.5 g/L of ammonium tartrate, 0.5 mL of 1% ferric citrate, 50 µg/L of thiamine, 0.23 g/L of casein hydrolysate, 0.5 g/L of yeast extract, and 10 g/L of glucose. The agar was removed from the modified PDA or MNC medium to prepare liquid spawn, which was incubated at 22 °C in a thermostatic oscillator for 20 days at a speed of 150 r/min.

**Table 1:** Fungal strains

Strain number	Species	Origin	GenBank number
S1	*Suillus bovinus*	Kunyushan	OM846602
S2	*Suillus luteus*	Kunyushan	OM846601
S3	*Suillus grevillei*	Xiaoyuanmiao	OM865366
R1	*Retiboletus sinensis*	Xiaoyuanmiao	OL339344

### Host plant seedling preparation

*P. thunbergii* and *Q. acutissima* fruits were collected from Ludong Mountain near the Ludong University campus. Seeds were extracted from the ripe fruits and rinsed with sterile water for 4 h. Next, they were soaked in 1 mg/L of GA3 solution for 2 d, and the water solution was changed every 8 h in a refrigerator to break dormancy. The seeds were used immediately after treatment to prevent damage during long-term storage. After soaking, non-dormant seeds were transferred to an antiseptic solution containing sodium hypochlorite (2% available chlorine) and 0.01% polyoxyethylene sorbitan monooleate (Tween 80) (*P. thunbergii* and *Q. acutissima* seeds were soaked for 0.5 h and 2 h, respectively). After a thorough rinse in sterile water, the seeds were sown separately in autoclaved substrates composed of vermiculite and water (1:1 by volume) in large, sterilised glass jars in April 2021.

### Inoculation methods

One month after sowing, the healthy, vigorous seedlings were transferred to the bottom of straight-sided, polycarbonate-based, 500-mL wide-mouth transparent autoclaved jars that contained a vermiculite, perlite, and vermiculite substrate (2:1:1 by volume) [24, 25], followed by inoculation with fungi. Seedlings of each tree species were inoculated with 100 mL of liquid spawn per plant. All jars were fitted with four aeration holes sealed with a fluorocarbon membrane filter (pore size, 0.45 μm). The seedlings were axenically given 10–20 mL of deionised water per month to compensate for water loss owing to evaporation.

### Plant morphology and physiology assessments

#### Mycorrhizal infection rate

The mycorrhizal infection rate was determined by the grid-crossing method after six months of cultivation using the three inoculation methods. The mycorrhizal length was denoted as ‘a’, and the root length as ‘b’; the mycorrhizal infection rate (‘c’) was calculated as c = a / b [26].

### Photosynthetic parameters

Twelve months after mycorrhizal synthesis (June 2022), the photosynthetic rates and photosynthetic pigments of the seedlings were measured using a photosynthetic rate instrument.

### Plant hormone assessments

Plant hormones were assessed using the methods of Yin and Qi [27] with some modifications.

For the mushroom fermentation broths, 20 mL of fermentation solution was extracted via pipette from each type of boletes. A cold 80% methanol aqueous solution (stored at 3 °C) was added, and the mixture was stored at 3 °C for 8 h.

For the plant samples, the roots, stems, and leaves of *P. thunbergii* and *Q. acutissima* were washed in running water for 30 min and then three times with pure water to remove any residue. Then, 2 g each of the fresh roots, stems, and leaves were ground using a mortar in liquid nitrogen. The ground plant material was combined to create one 6-g sample for each species. A cold 80% methanol aqueous solution (stored at 3 °C) was added, and the mixture was refrigerated at 3 °C for 8 h.

The pre-treated samples were centrifuged at 4 °C and 8500 × g for 10 min, then the supernatant was collected in a new centrifuge tube and stored at 3 °C. The remaining precipitate was extracted with an 80% methanol aqueous solution for 4 h, followed by centrifugation at 4 °C and 8500 × g for 10 min. The supernatant was collected and combined with that from the first centrifugation. A nitrogen blow dryer was used to remove the methanol from the supernatant, and then the remaining liquid was combined with petroleum ether in a separation funnel for extraction and decolourisation three times. The pH of the solution was adjusted to 3 using 1 mol/L of citric acid. Then, three rounds of ethyl acetate extractions were performed. The organic extracts from the three extractions were pooled, and a nitrogen blow dryer was used to dry the ethyl acetate. Finally, the extract was dissolved in 1 mL of methanol and filtered through a 0.22-µm needle organic filter for high-performance liquid chromatography (HPLC) analyses.

A ZORBAX SB-C18 (4.6 mm × 250 mm, 5 μm; Agilent Technologies, Santa Clara, CA, USA) chromatographic column and methanol-formic acid gradient elution mobile phase were used. The flow rate was 1.0 mL/min, the column temperature was 25 °C, the injection volume was 20 µL, and the detection wavelengths were 260 and 220 nm. Table 2 details the HPLC gradient elution system.

**Table 2:** High-performance liquid chromatography gradient elution system

Time	Methanol	0.06% Formic acid
0–15 min	48.7%	51.3%
15–20 min	50.7%	49.3%
20–30 min	50.8%	49.2%

### Statistical analyses

The statistical analysis was conducted using SPSS version 19 (IBM Corp., Armonk, NY, USA) and GraphPad Prism 9 (GraphPad Inc., San Diego, CA, USA) software. Variance analyses were implemented within the experiments. Ten replicates were performed per experimental group, and each group was repeated three times.

## Results

### Internal transcribed spacer (ITS) sequence alignment and pure culture of boletes

Mycelia of the four bolete species were obtained through tissue isolation and purification. After two months of mycelial culture, ITS sequences were acquired by amplifying the DNA of the mycelium, followed by BLAST alignment in GenBank. The S1 (OM846602), S2 (OM846601), and R1 (OL339344) ITS sequences showed 99.69%, 99.32%, and 100% homology with those of the Chinese *S. bovinus*, *S. luteus*, and *R. sinensis* specimens, respectively [28–30]. The S3 ITS sequence (OM865366) showed 99.03% homology with that of Japanese *S. grevillea* [31]. Original fruiting bodies were sampled from mixed coniferous and broad-leaved forests in Yantai, China, at the Xiaoyuanmiao Forest Farm and Trapped Mountain Forest Farm (Table 1). A representative strain was registered at the China General Microbiological Culture Collection Center (CGMCC No. 23,887).

**Fig. 1:**
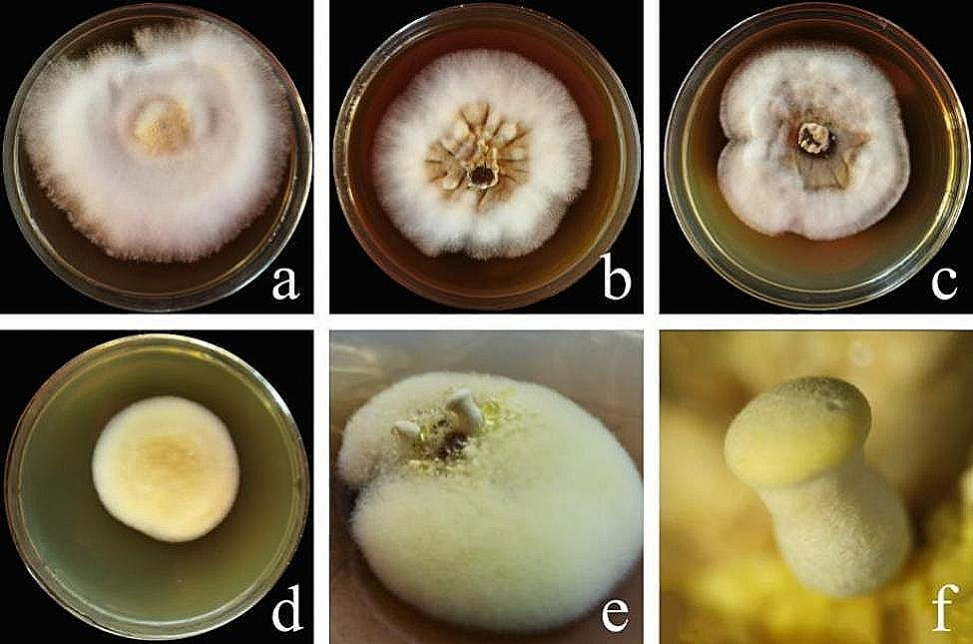
Mycelia of the (**a**) *Suillus bovinus*, (**b**) *Suillus luteus*, (**c**) *Suillus grevillea*, and (**d**) *Retiboletus sinensis* bolete species and the **e**, **f**) fruiting body of *R. sinensis*

The three *Suillus* species had similar mycelial morphologies. The mycelia were white and dense, primarily comprising creeping mycelia that secreted brown substances during the later stages of culture (Fig. 1a–c). The mycelia of *R. sinensis* were light yellow, mainly comprising erect mycelia, which secreted brown substances during the later stages of culture. During the pure culture process, the mycelia were stimulated by temperature and scattered light, producing primordium and fruiting bodies independent of the symbiotic plants. The primordium of the fruiting body was light yellow and millet grain-shaped. The pileus was light yellow during the early stages of fruiting body differentiation, and the stipe was white with villi (Fig. 1d–f).

### Mycorrhiza appearance and morphology

**Fig. 2:**
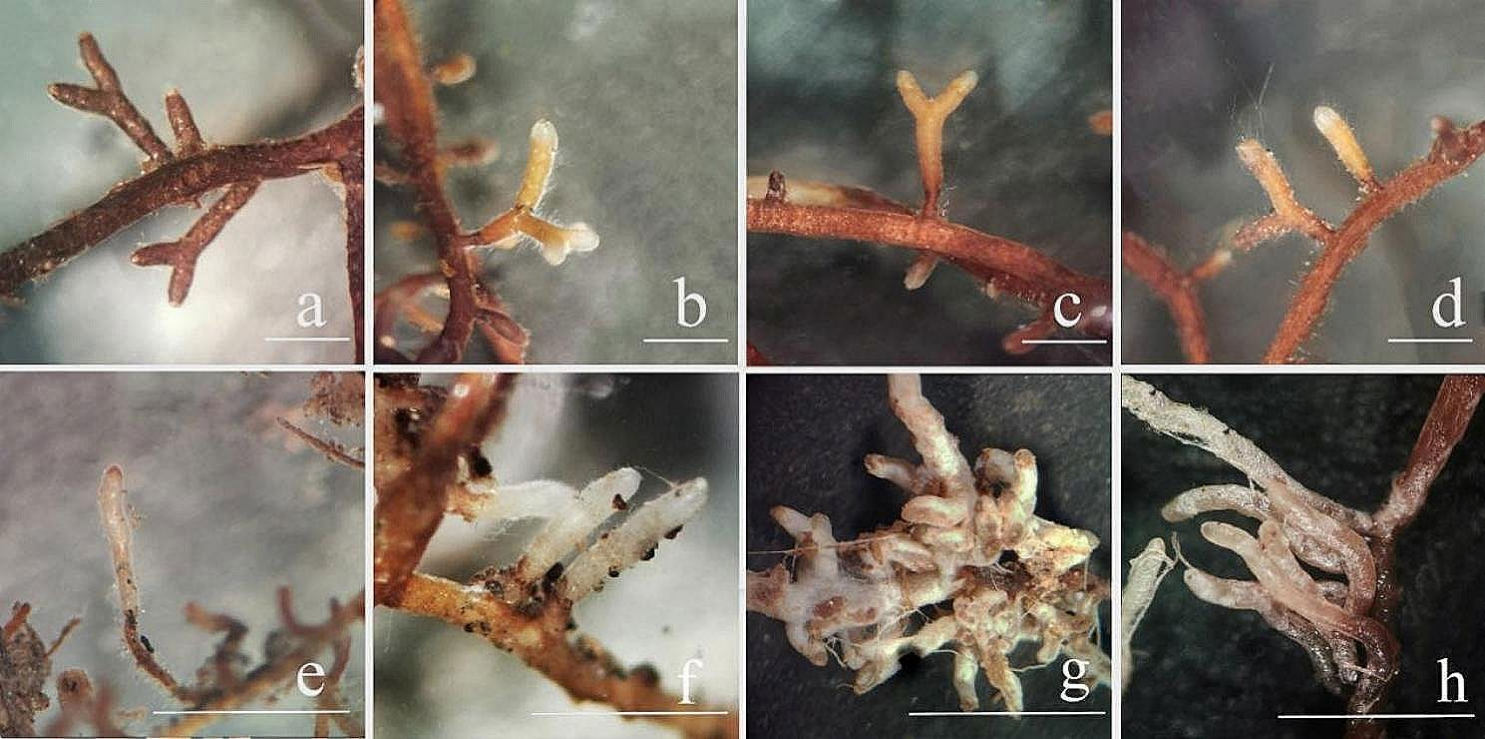
Mycorrhizae morphology: a–d) *Pinus thunbergii* with (**a**) *Suillus bovinus*, (**b**) *Suillus luteus*, (**c**) *Suillus grevillea*, and *Retiboletus sinensis*; **d**–**h**) *Quercus acutissima* with **e**) *S. bovinus*, **f**) *S. luteus*, **g**) *S. grevillea*, and **h**) *R. sinensis*

The appearances of the bolete mycorrhizae were observed using a stereoscope. Combining each mushroom species with *P. thunbergii* (Fig. 2a–d) predominantly resulted in mycorrhizae with a bifurcate branch structure, but a small amount was rod-like, which was likely an unbranched or soon-to-be bifurcated binary-branching structure. A small number of hyphae were observed on the surface of the mycorrhizae, and there was no apparent fungal sheath. The mycorrhizal branches were 0.6–1.23 mm long and 0.22–0.29 mm thick.

**Fig. 3:**
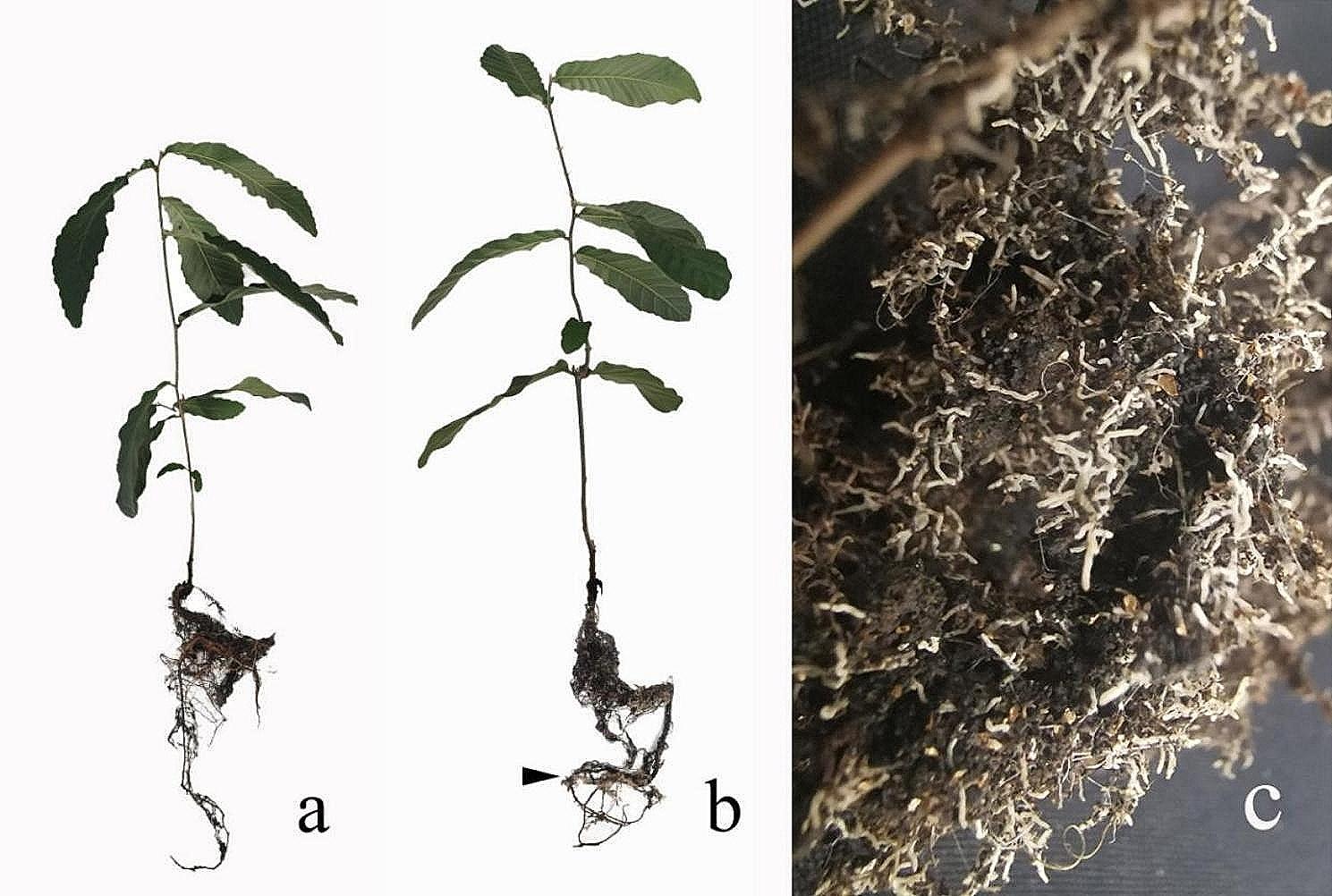
*Quercus acutissima* seedling morphology (**a**) without and (**b**) with ectomycorrhizae inoculation. (**c**) Mycorrhiza from *Q. acutissima*

**Fig. 4:**
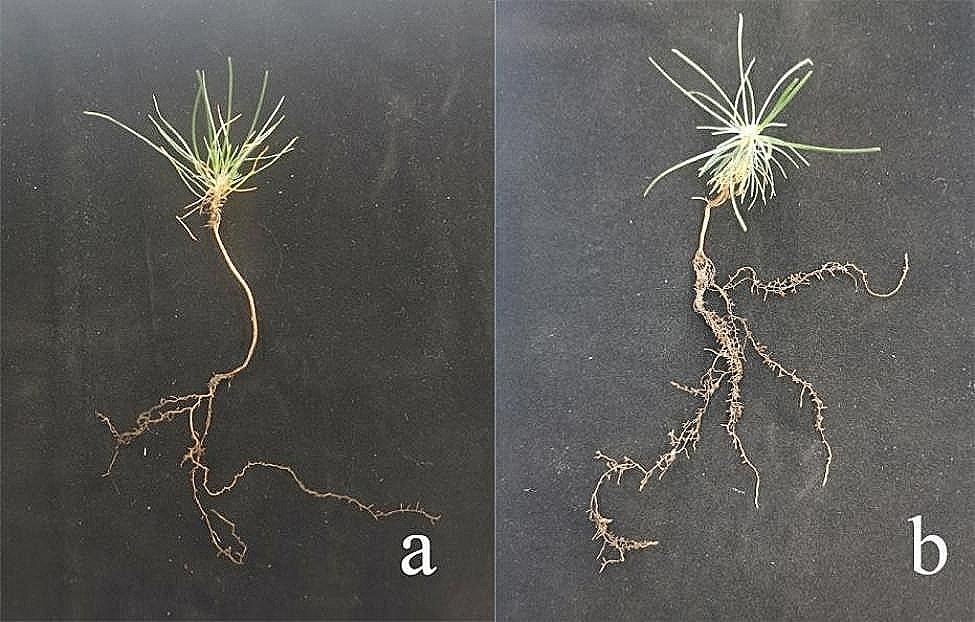
Morphology of (**a**) non-mycorrhizal and (**b**) mycorrhizal *Pinus thunbergii* seedlings

Combining each mushroom species with *Q. acutissima* (Fig. 2e–h) resulted in only rod-like structures; bifurcated branched structures were not observed. The hyphae on the surfaces of the mycorrhizae were visibly entangled. The mycorrhizae were 0.33–0.96 mm long and 0.05–0.18 mm thick. The rod-shaped mycorrhizae of *Q. acutissima* were significantly smaller than those of *P. thunbergia*. However, the mycorrhizal density of *Q. acutissima* was much higher than that of *P. thunbergii* (Figs. 3 and 4).

The *S. bovinus* and *S. luteus* mantles were not obvious, and the mycelia on the mycorrhizal surface were sparse. In contrast, the *S. grevillea* and *R. sinensis* mantles were very pronounced, and their roots were fascicled.

Thus, the rod-like or bifurcated structures of the mycorrhizae of these four bolete species were determined based on the symbiotic plant rather than the bolete species. The mycorrhizal morphologies of the bolete species also slightly differed.

The mycorrhizal infection rates for *S. bovinus*, *S. luteus*, *S. revillea*, and *R. sinensis* were 49.7%, 54.97%, 52.3%, and 43.4% for *P. thunbergia* and 54.83%, 59.6%, 48.43%, and 55.37% for *Q. acutissima*, respectively.

### Host plant appearance and biomass

**Fig. 5:**
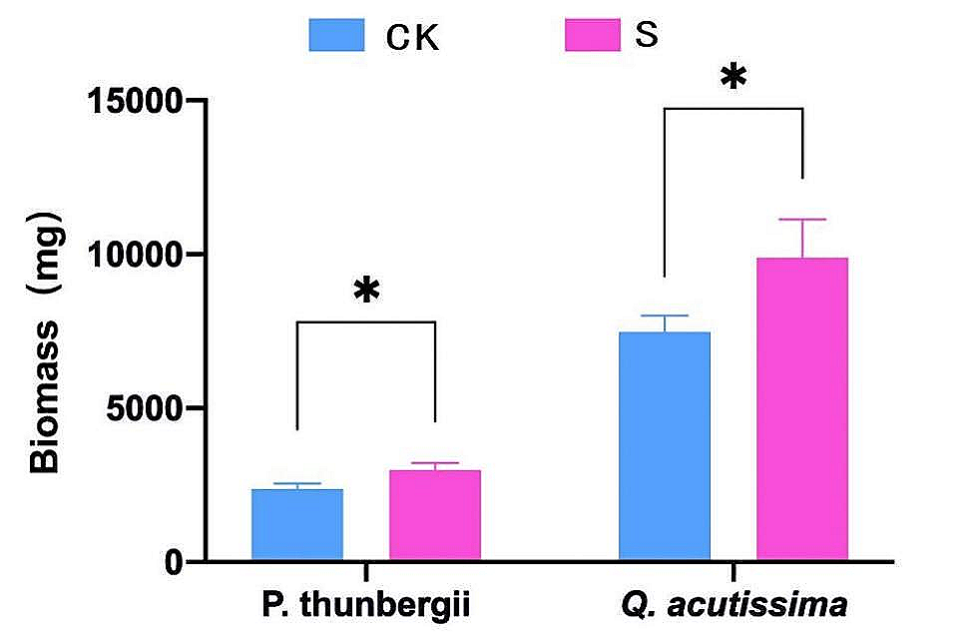
Host plant (*Pinus thunbergii* and *Quercus acutissima*) biomass. CK, non-mycorrhizal plants; S, mycorrhizal plants. * *p* < 0.05

Inoculation changed the appearance of both host plants. The inoculated *P. thunbergii* and *Q. acutissima* seedlings had significantly more root development and lateral roots than the non-inoculated seedlings. The biomass of the inoculated *P. thunbergii* and *Q. acutissima* seedlings also significantly increased compared to the non-inoculated seedlings (Fig. 5), increasing by 25% for *P. thunbergii* and 32% for *Q. acutissima.*

### Photosynthetic parameters

**Fig. 6:**
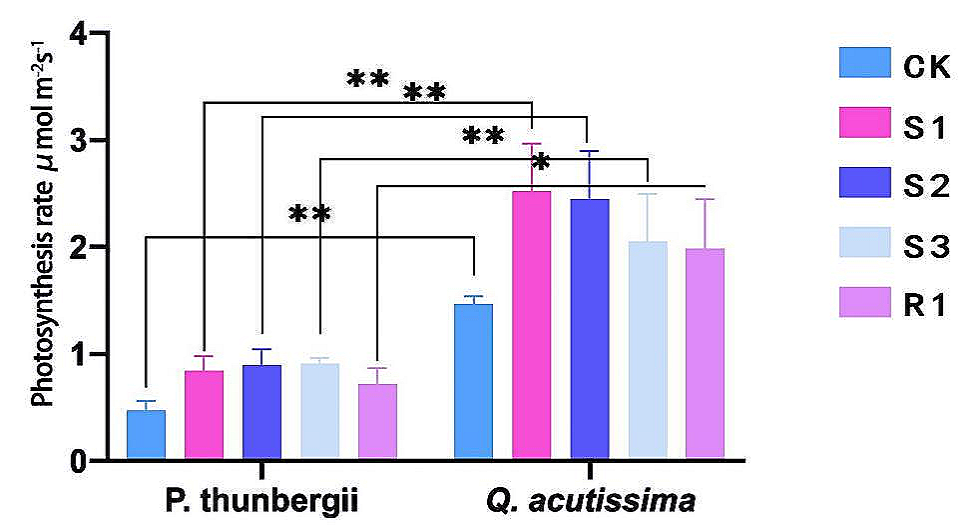
Photosynthetic rates of *Pinus thunbergii* and *Quercus acutissima*

Inoculated *P. thunbergii* and *Q. acutissima* seedlings had significantly higher photosynthetic rates than the non-inoculated seedlings (*p* < 0.05; Fig. 6). The photosynthetic rate increase for *P. thunbergii* ranged from 51 to 93%, increasing the most (by 93%) after inoculation with *R. sinensis* and the least (by 51%) with *S. grevillea* compared to the non-inoculated group. The photosynthetic rate increase for *Q. acutissima* ranged from 35 to 72%, increasing the most (by 72%) with *S. bovinus* and the least (by 35%) with *R. sinensis* compared to the non-inoculated group.

CK: Non-inoculated *P. thunbergii* or *Q. acutissima* (control). S1: inoculated with *Suillus bovinus*; S2: inoculated with *Suillus luteus*; S3: inoculated with *Suillus grevillea*; and R1: inoculated with *Retiboletus sinensis*. * *p* < 0.05; ** *p* < 0.01.

### Plant hormone method assessments

The six plant hormones measured in this study exhibited excellent linearity within their respective linear ranges, with correlation coefficients no lower than 0.9980 (Table 3). The intra-day and inter-day precisions were lower than 0.4% and 2.5%, respectively. The detection limits fell between 0.026 and 0.043 µg/mL. These results demonstrate that the methodology used in this study has high repeatability, a relatively low limit of detection, and acceptable precision and sensitivity.

**Table 3:** Parameters associated with the plant hormone assessment method

Hormone	Regression equation	Linear range (µg/mL)	R^2^	Precision (%) (*n* = 5)		Detection limit (µg/mL)
				Intra-day	Inter-day	
IAA	Y = 78.032X– 2.501	0.5–80	0.9993	0.32	1.98	0.032
ZT	Y = 71.834X − 9.2509	0.25–40	0.9991	0.26	2.03	0.028
GA	Y = 59.963X– 20.991	0.25–40	0.9989	0.33	2.25	0.026
ABA	Y = 31.913X– 2.984	0.5–80	0.9996	0.21	2.08	0.043

IAA: indole-3-acetic acid; ZT: zeatin; GA: gibberellic acid; ABA: abscisic acid.

### Bolete fermentation broth hormone content

**Fig. 7:**
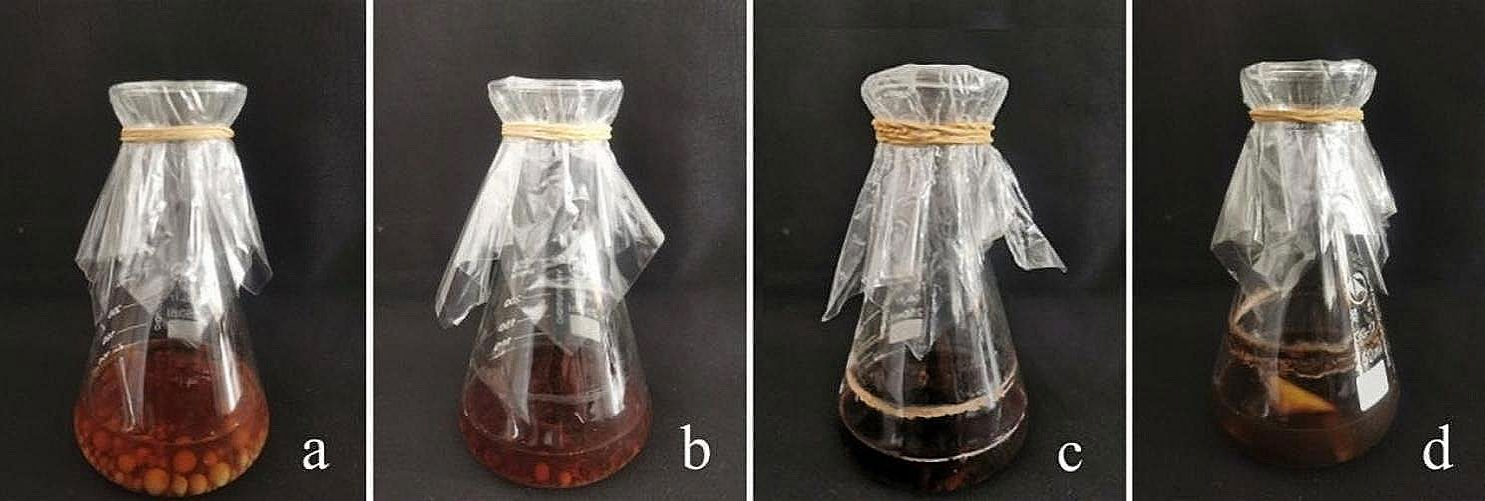
Liquid strains of (**a**) *Suillus bovinus*, (**b**) *Suillus luteus*, (**c**) *Suillus greville*, and (**d**) *Retiboletus sinensis*

**Fig. 8:**
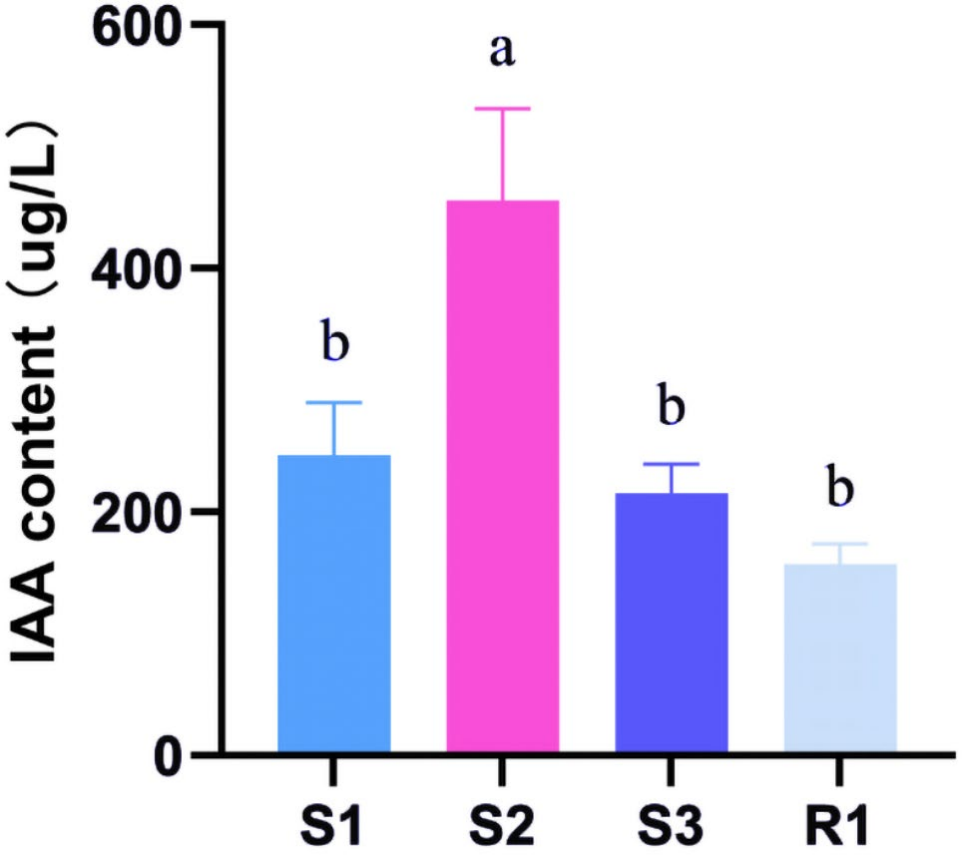
Indole-3-acetic acid (IAA) content of *Suillus bovinus* (S1), *Suillus luteus* (S2), *Suillus grevillea* (S3), and *Retiboletus sinensis* (R1) fermentation broths. a: definition; b: definition

**Fig. 9:**
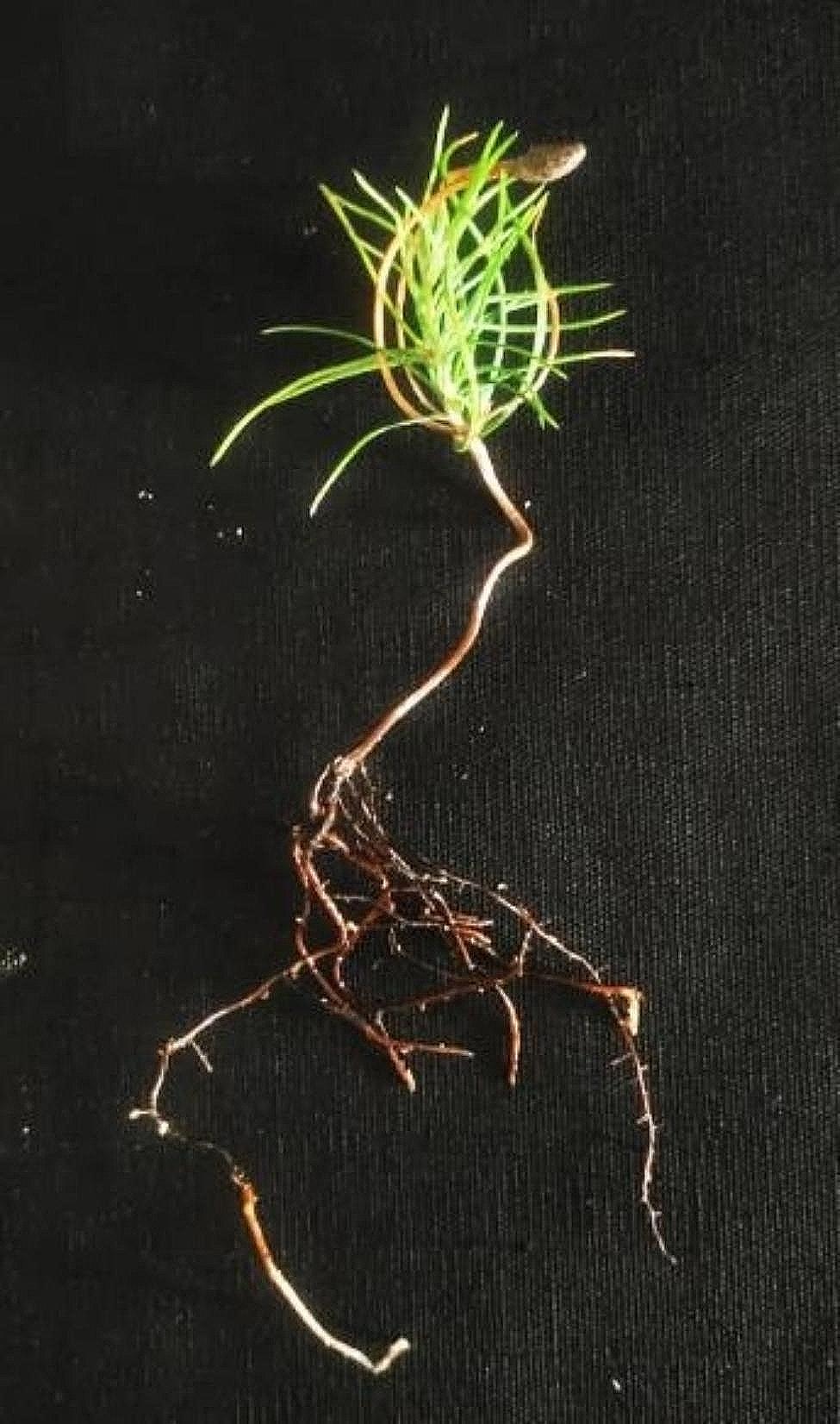
*Pinus thunbergii* seedling without mycorrhizal formation after inoculation with [insert]

After 20 d of liquid fermentation of the mycelia, the fermentation broths of all mushroom species were brown to dark brown with a strong aroma (Fig. 7). The fermentation broth had a high level of IAA, but zeatin (ZT), gibberellic acid (GA), and abscisic acid (ABA) were not detected. The *S. luteus* broth had the highest IAA level (455.77 µg/L), followed by 246.67 µg/L in the *S. bovinus* broth, 215.39 µg/L in the *S. grevillea* broth, and 157.25 µg/L in the *R. sinensis* broth. The IAA content did not differ among the *S. bovinus*, *S. grevillea*, and *R. sinensis* fermentation broths (one-way analysis of variance; Fig. 8). The root changes in *Q. acutissima* seedlings after irrigation with the fermentation broth were comparable to the effects of IAA in the tissue culture (Fig. 9).

### Seedling hormone contents

**Fig. 10:**
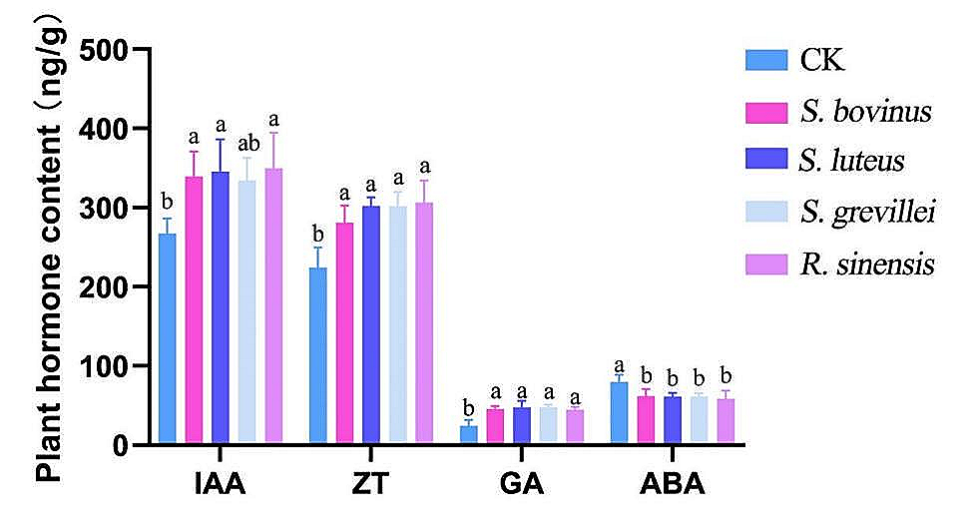
Hormone content of *Quercus acutissima* seedlings inoculated with four bolete species. CK, non-inoculated *Q. acutissima* seedlings; IAA, indole-3-acetic acid; ZT, zeatin; GA, gibberellic acid; ABA, abscisic acid. a: definition; b: definition

The IAA contents in the inoculated *Q. acutissima* seedlings increased by 27.1% (*S. bovinus*), 29.5% (*S. luteus*), 25.2% (*S. grevillei*), and 30% (*R. sinensis*) compared to the non-inoculated seedlings (Fig. 10). Similarly, the total ZT content of inoculated seedlings increased by 25.4%, 34.8%, 34.6%, and 36.7%, respectively. Of the four hormones, the GA content exhibited the most significant change, with the total GA content increasing by 86.3%, 95.1%, 95.7%, and 83.1% for *S. bovinus*, *S. luteus*, *S. grevillei*, and *R. sinensis*, respectively, compared to non-inoculated seedlings. In contrast, the ABA content was lower in the inoculated seedlings than in the non-inoculated seedlings, with the total ABA content decreasing by 22.7%, 23.3%, 23.1%, and 26.8% for *S. bovinus*, *S. luteus*, *S. grevillei*, and *R. sinensis*, respectively.

The total GA to ABA (GA/ABA) ratio for all inoculated seedlings was significantly higher than that in the sterile root seedlings (*p* < 0.05). The GA/ABA ratio in the sterile root seedlings was 0.31, whereas the ratios in the *S. bovinus*, *S. luteus*, *S. grevillei*, and *R. sinensis* mycorrhizal seedlings were 0.74, 0.78, 0.78, and 0.76, respectively; the ratios did not differ among the groups. The IAA/ABA ratios of *S. bovinus*, *S. luteus*, *S. grevillei*, and *R. sinensis* mycorrhizal seedlings were 5.50, 5.64, 5.44, and 5.98, respectively, which were significantly higher than the ratio (3.34) observed for the sterile seedlings. The ZT/ABA ratios in *S. bovinus*, *S. luteus*, *S. grevillei*, and *R. sinensis* mycorrhizal seedlings were 4.55, 4.93, 4.91, and 5.24, respectively, which were also significantly higher than the ratio (2.81) observed for the sterile seedlings. The IAA/GA ratios for *S. bovinus*, *S. luteus*, *S. grevillei*, and *R. sinensis* mycorrhizal seedlings were 7.46, 7.36, 6.99, and 7.83, respectively, which were significantly lower than the ratio (10.95) for the aseptic seedlings. Finally, the IAA/ZT ratios for the *S. bovinus*, *S. luteus*, *S. grevillei*, and *R. sinensis* mycorrhizal seedlings were 1.21, 1.44, 1.11, and 1.14; these ratios did not differ from the ratio observed in sterile root seedlings (1.19).

**Fig. 11:**
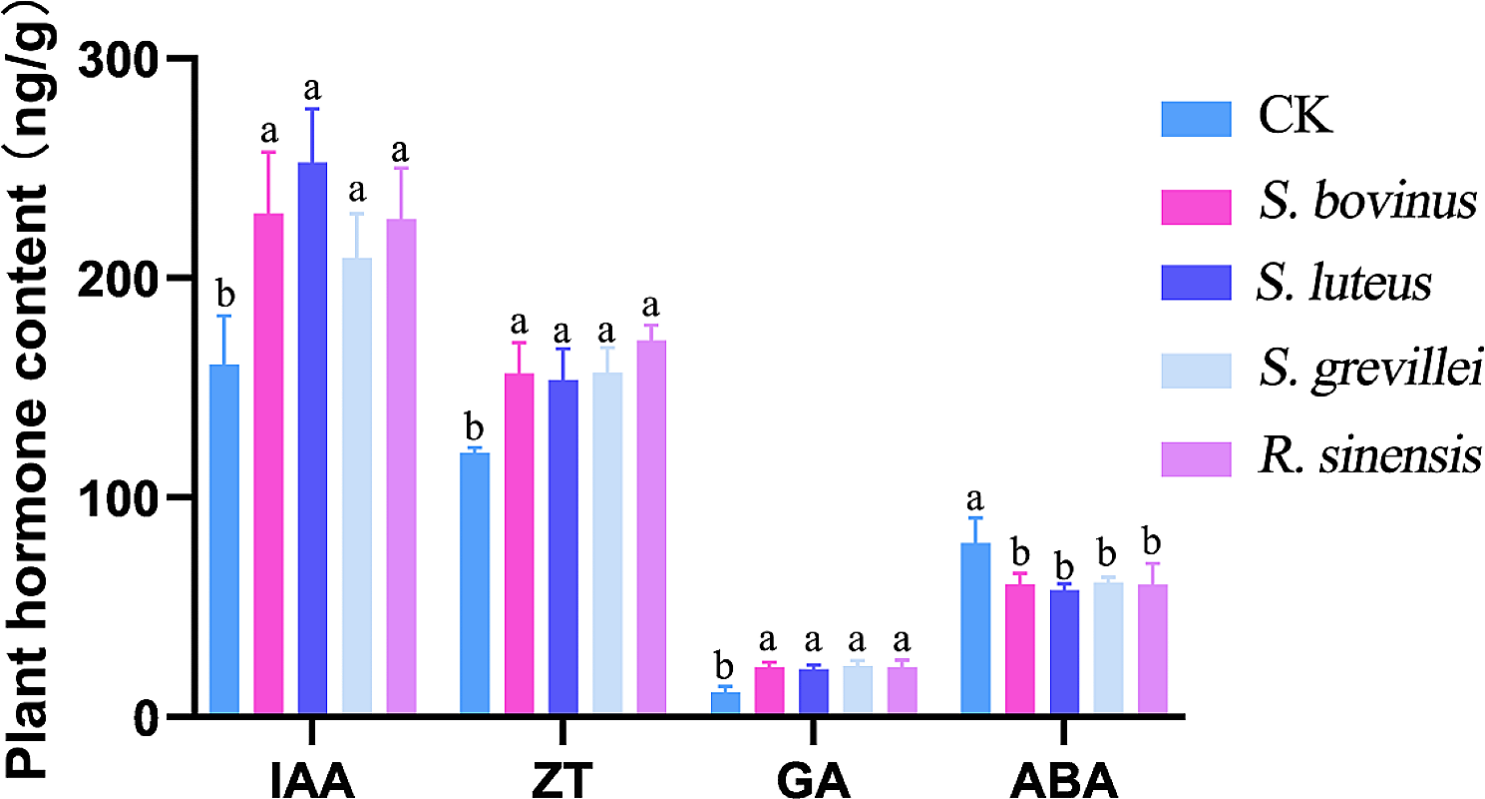
The hormone content of *Pinus thunbergii acutissima* seedlings inoculated with four bolete species. CK, non-inoculated *P. thunbergii* seedlings (control); IAA, indole-3-acetic acid; ZT, zeatin; GA, gibberellic acid; ABA, abscisic acid. **a**: definition; **b**: definition

The IAA, ZT, and GA contents increased, and the ABA content decreased in the *P. thunbergii* seedlings inoculated with the four bolete species compared to the non-inoculated seedlings (Fig. 11). The IAA content in the inoculated seedlings increased by 28.7% (*S. grevillei*), 30.8% (*S. bovinus*), 29.8% (*R. sinensis*), and 33% (*S. luteus*) compared to the non-inoculated seedlings. Furthermore, the total ZT contents in the *S. bovinus*, *S. luteus*, *S. grevillei*, and *R. sinensis* inoculated seedlings increased by 28.1%, 28.05%, 38.5%, and 42.5%, respectively, and the total GA contents increased by 98.1%, 90%, 102.8%, and 102%, respectively. In contrast, the ABA contents of the *S. bovinus*, *S. luteus*, *S. grevillei*, and *R. sinensis* inoculated seedlings were significantly lower than those of the control group; the total content decreased by 24.1%, 26.9%, 22.6%, and 29%, respectively.

The total GA/ABA ratio for all inoculated seedlings was significantly higher than that in the sterile root seedlings (*p* < 0.05). The GA/ABA ratio was 0.14 for the sterile root seedlings and 0.38 for the mycorrhizal seedlings, with no significant difference. The IAA/ABA ratios of the *S. bovinus*, *S. luteus*, *S. grevillei*, and *R. sinensis* inoculated seedlings were 3.43, 3.62, 3.39, and 3.53, respectively, which were significantly higher than those of the non-inoculated seedlings (2.02). The ZT/ABA ratios of the *S. bovinus*, *S. luteus*, *S. grevillei*, and *R. sinensis* inoculated seedlings were 2.56, 2.66, 2.55, and 2.84, respectively, which were significantly higher than that for the control seedlings (1.52). The IAA/GA ratios of the *S. bovinus*, *S. luteus*, *S. grevillei*, and *R. sinensis* inoculated seedlings were 8.42, 9.52, 8.79, and 8.31, respectively, which were significantly lower than those of the non-inoculated seedlings (15.96). Finally, the IAA/ZT ratio of the *S. bovinus*, *S. luteus*, *S. grevillei*, and *R. sinensis* inoculated seedlings were 1.34, 1.36, 1.32, and 1.24, respectively, which did not differ from the non-inoculated seedling ratio (1.33).

The bolete mushrooms elicited growth-promoting effects on the pine and oak seedlings, significantly altering the growth hormone levels of the seedlings after inoculation. Specifically, the IAA, ZT, and GA levels increased, whereas the ABA levels decreased. These results suggest that bolete mushrooms form symbiotic relationships with plant seedlings, promoting plant growth. The GA/ABA, IAA/ABA, and ZT/ABA ratios all increased significantly compared to the non-inoculated seedling ratios, implying that the ratio of growth-promoting hormones (GA, IAA, and ZT) to growth-inhibiting hormones (ABA) increased, potentially enhancing the seedlings’ growth rate.

In mycorrhizal seedlings, the IAA/GA values significantly decreased, which may help regulate plant growth and maintain a balance between growth rate and structural development. However, the IAA/ZT ratios did not differ between the two tree species, indicating that the relative proportions of growth hormones and cytokinins remained stable.

## Discussion

This study is the first to explore the secretion of indole compounds from the fermentation broths of four bolete species and to use sterile *P. thunbergii* and *Q. acutissima* seedlings to form mycorrhiza. This report describes the appearance of the mycorrhiza in detail and examines physiological changes in symbiotic plants. Notably, we found that *R. sinensis* produces fruiting bodies under pure culture conditions, which is a significant finding in the field of bolete cultivation and provides valuable information for studying the artificial cultivation of boletes. Our future studies will investigate this species further.

Previous studies have found that ECMFs secrete indole compounds, which strengthen the effect of the fungi on root development, particularly by promoting lateral root formation and the creation of more mycorrhizas. IAA secreted by ECMFs helps form the Hartig network as a diffusion signal [32, 33]. In this study, we measured the IAA contents in the fermentation broths of four boletes, which confirmed our hypothesis that these boletes secrete IAA to participate in the mycorrhizal colonisation process, similar to other reported ECMFs. Many microorganisms capable of secreting IAA and IAA precursors form beneficial relationships with plants and alter host root development. Furthermore, other signals produced by microorganisms affect the hormone contents of host plants [34].

In this study, four bolete species formed mycorrhizal symbionts with *P. thunbergii* and *Q. acutissima*. *Q. acutissima* mycorrhiza was predominantly rod-shaped, while *P. thunbergii* mycorrhiza primarily exhibited a binary branching structure (Fig. 5). These results generally agree with those reported by Wang et al. [35]. This binary branching structure appears to be an inherent feature of pine plants [36–38], likely resulting from changes in plant hormones caused by fungal secretion of hormones or other signals [39, 40]. Although the specific reasons for this change remain unclear, this may explain why the roots of the inoculated seedlings were more developed than those of the control group (Figs. 6 and 7).

ECMFs are of considerable ecological importance, particularly in areas with poor site conditions and fragile ecological environments. Seedling growth quality directly determines the success of afforestation, and growth indicators reflect seedling quality more intuitively. Studies have found that most woody plants in northern temperate forests form mutually beneficial symbionts with ECMFs, exchanging nutrients and water for carbon fixed by photosynthesis [41]. Owing to the prevalence of this symbiotic interaction, ECMFs may play a critical role in ecosystem restoration and regulation [42]. However, the improvement in plant nutrition caused by this interaction is costly. Mycorrhizas absorb approximately half of their photosynthetic products, and plants meet the high-carbohydrate requirements of mycorrhizal symbionts by enhancing their photosynthetic intensity [43]. In the present study, all four bolete species increased the photosynthetic rate of the *P. thunbergii* and *Q. acutissima* seedlings by 51–93%, consistent with results presented by Pengfei et al. [44].

We also determined the IAA, ZT, GA, and ABA contents in the roots, stems, and leaves. IAA, primarily indole-butyric acid, promotes robust plant growth [45, 46], and we found increased IAA content in the roots, stems, and leaves of the plants, with the highest concentration in the roots. When fungi promote plant growth, they initially help plants develop their roots. As mentioned, IAA secreted by fungi assists plants in forming more lateral roots, which helps them absorb more nutrients and water. ZT regulates the opening and closing of plant stomata and controls the photosynthesis rate [47]. In this study, the ZT contents in the roots, stems, and leaves of the inoculated plants increased to varying degrees. Although the ZT contents in some stems and leaves did not differ from those in the control group, fungal invasion affected the ZT content in their symbiosis, affecting the synthesis of photosynthesis-related organic matter. This may be one explanation for the change in the photosynthetic rate mentioned above, which is consistent with the findings of Yin & Qi [27].

A synergistic effect between GA and IAA regulates the IAA content, prevents organ detachment, and breaks seed dormancy [48]. GA regulates various essential plant growth and development processes, including photosynthesis [49]. In this study, the GA contents changed the most in the leaves and the least in the roots and stems of both host species, which also increased the photosynthetic rates of those plants inoculated with fungi. ABA promotes plant root growth, a solid underground root system to overcome survival challenges under environmental stress, and an increased root absorption area [50, 51]. In this study, the ABA contents in the roots, stems, and leaves of both tree species inoculated with all bolete species were significantly lower than those of the control group. Therefore, the hormone content in the plants changed significantly after bolete inoculation, which directly or indirectly changed the morphology and biomass of the symbiotic plants.

## Conclusions

This study investigated the physiological effects of four boletes on tree seedlings from the perspective of plant growth regulators. In the future, we will explore related gene expression and signal transduction. In summary, this study provides foundational data for future research and a theoretical basis for applied ectomycorrhizal science.

## Data Availability

The datasets analysed during the current study are available from the corresponding author upon reasonable request.
